# Rapid Thromboelastography Identifies Coagulopathy and Predicts Poor Outcomes in Severe Traumatic Brain Injury

**DOI:** 10.7759/cureus.87710

**Published:** 2025-07-11

**Authors:** Tushar Sehgal, Tapasyapreeti Mukhopadhyay, Chandan Mishra, Anand Kumar, Arulselvi Subramanian, Deepak Agrawal, Geetha Menon

**Affiliations:** 1 Laboratory Medicine, All India Institute of Medical Sciences, New Delhi, IND; 2 Neurosurgery, All India Institute of Medical Sciences, New Delhi, IND; 3 Biostatistics, Indian Council of Medical Research, New Delhi, IND

**Keywords:** coagulopathy, mortality, rapid thromboelastography, severe traumatic brain injury, viscoelastic assays

## Abstract

Introduction

Traumatic brain injury (TBI) occurs when a force transmitted to the head or body results in neuropathologic damage and dysfunction. Approximately 25% of patients with TBI present with coagulopathy on admission, which is associated with increased mortality. Viscoelastic methods like rapid thromboelastography (r-TEG) and rotational thromboelastometry may be precise in identifying the coagulopathic changes in these patients. The objective of this study is to assess r-TEG in patients with severe TBI (sTBI).

Methods

This was a single-center cross-sectional study conducted in a 2059-bedded level 1 trauma center. Patients over 18 years of age who presented with sTBI [Glasgow Coma Scale (GCS), ≤8] with head injuries were included. All clinical and laboratory data were obtained from the charts. r-TEG was done according to the manufacturer’s protocol by a single operator. The coagulopathy was categorized as hypercoagulable, hypocoagulable, and normal based on r-TEG variables. Laboratory parameters and clinical outcomes were compared between the three groups.

Results

One hundred five patients with a median age of 33 years [interquartile range (IQR), 25-40 years] were included. The majority of patients (91%) were male. The commonest mechanism of injury was a road traffic accident (RTA) in 75% cases. The coagulopathy was hypercoagulable in six (6%), hypocoagulable in 38 (36%), and normal in 61 (58%) patients. The overall mortality rate was 36%. Among the r-TEG parameters, reaction (R) time, kinetic (K) time, alpha angle, maximum amplitude (MA), thrombodynamic potential index (TPI), time to maximum amplitude (TMA), shear elastic modulus (G), elasticity (E), and amplitude (A) were statistically significant between all groups. The age-adjusted multivariate analysis showed the following clinical and laboratory parameters as predictors of mortality: GCS, systolic blood pressure (SBP), blood sugar, aPTT, fibrinogen, R-time, K-time, alpha angle, and activated clotting time (ACT). The 57-day mortality among patients with hypocoagulable and hypercoagulable was higher than that of patients with normal coagulation status (log-rank test, p = 0.35)

Conclusion

r-TEG identifies coagulopathy in patients with sTBI. The mortality was higher in patients with coagulopathy than in those with a normal coagulation state.

## Introduction

Hemorrhage is responsible for up to 40% of trauma deaths and increased morbidity in traumatic brain injury (TBI) [1]. TBI occurs when a force transmitted to the head or body results in neurological damage and dysfunction [2]. TBI accounts for 2.5 million emergency visits in the United States [3]. Among these patients, 25% are coagulopathic on admission, which is associated with a fivefold increase in mortality [4]. Current evidence shows that tissue injury, hypoperfusion, accelerated fibrinolysis, and inflammatory responses are responsible for trauma-induced coagulopathy (TIC) [1]. The severity and outcome of TBI can be quantified using a simple and standardized Glasgow Coma Scale (GCS) score [2]. The GCS is used to grade TBI as mild, moderate, or severe [2]. Mild TBI (mTBI) is the most common (75-85%) with a GCS score of 13-15.5. mTBI includes concussion as well as sub-concussion and may occur in sports activities, military service, and in association with poorly controlled epilepsy, head banging, and physical abuse [2]. There is often full neurologic recovery after mTBI; however, about one-third of subjects develop prolonged neurocognitive and behavioral changes [5,6]. In moderate TBI (GCS, 9-12), the patient is initially stuporous, and in severe TBI (sTBI) (GCS, 3-8), the patient is comatose, unable to open the eyes or follow commands. Patients with sTBI are at high risk for secondary brain injury, including hypotension, hypoxemia, and brain swelling [7]. In sTBI, there is a direct linear relation to a poor outcome, including severe neurologic disability, vegetative state, and death. Advancing age, over 60 years, is also associated with an increased risk of a poor outcome [7].

Coagulopathy in TBI has been related to the injury-mediated release of TF, activating the extrinsic pathway of coagulation. TF exists at high levels in the brain and is activated early in coagulopathy following head trauma [1]. TIC is identified by derangements in the conventional clotting tests (CCT) of prothrombin time, international normalized ratio (INR), activated partial thromboplastin time, and platelet counts, but CCT may not assess coagulopathy adequately [8]. Viscoelastic hemostatic assays (VHA), such as thromboelastography (TEG) and rotational thromboelastometry (ROTEM), are more precise in identifying the exact derangements of hemostasis compared to CCT. TEG has the ability to differentiate between enzymatic and platelet coagulopathy [9]. It provides information on clot initiation, clot growth, final clot strength, and presence of fibrinolytic clot breakdown, giving a global evaluation of all phases of the coagulation cascade [10]. VHA can guide resuscitation through real-time information about clot initiation, kinetics, strength, and dissolution, resulting in an expanded role in trauma [11-13]. 

Rapid TEG (r-TEG) is a modification of the classic TEG assay that utilizes TF instead of the kaolin-cephalin reagent to activate blood coagulation. As TF triggers the extrinsic coagulation pathway (with a smaller number of coagulation factors), the test can be performed faster than conventional TEG. r-TEG can be completed within 15 minutes and thus helps manage massive transfusions in trauma patients [14,15]. The objective of this study is to assess the use of r-TEG in sTBI.

## Materials and methods

Study setting

This was a single-center cross-sectional study conducted at a level 1 trauma center with 2059 beds from September 2021 to August 2024. The study was approved by the institutional ethical committee. Patients over 18 years of age who presented with sTBI, defined by an initial GCS of less than or equal to 8, were screened for inclusion. To be eligible for inclusion, the patients were required to have TEG and computed tomography (CT) available at baseline. The patients associated with extracranial injuries, clinical evidence of brain death, secondary admissions, and patients with a history of hemostatic products prior to TEG were excluded. 

Clinical and laboratory data

From the charts, the following information was extracted: demographics, blood pressure, heart rate, and GCS. The Injury Severity Score (ISS) was calculated from the Abbreviated Injury Scale (AIS), version 2008, from the Association for the Advancement of Automotive Medicine (AAAM) [16]. Whole blood was withdrawn within 24 hours of injury, prior to any fluid/blood transfusion by the phlebotomist in the emergency department. The blood counts were obtained from EDTA [BD Vacutainer® plastic tubes of 3 mL (buffered K2 EDTA 5.4 mg)] anticoagulated blood samples run on a Sysmex hematology analyzer (XN-9000), Kobe, Japan. CCT were performed on STA R Max®3 (Diagnostica Stago, France) using citrate vials [BD Vacutainer® plastic citrate tubes of 2.7 mL (0.109M, 3.2% buffered sodium citrate)] and included PT (STA®-NeoPTimal), INR, aPTT (STA®-Cephascreen), fibrinogen (STA®-Liquid Fib), and D-dimer (STA®-Liatest D-dimer). Data of serum creatinine (CREJ2, Roche Diagnostics, Indianapolis, IN, USA), serum urea (Ureal, Roche Diagnostics), total bilirubin (BILT3, Roche Diagnostics), blood glucose (GLUC3, Roche Diagnostics), and electrolytes (sodium, potassium, and chloride) (ISE reference, Roche Diagnostics) were obtained from serum samples collected in BD Vacutainer® SST tubes and run on a Cobas c701 automated chemistry analyzer (Roche Diagnostics).

Protocol for r-TEG

All patients underwent both r-TEG after enrolment into the study. r-TEG was done according to the manufacturer’s protocol by a single operator. Blood was collected in citrate vials [BD Vacutainer® plastic citrate tubes of 2.7 mL (0.109 M, 3.2% buffered sodium citrate)], and r-TEG was run within four hours of sample collection. It was performed by automated TEG 5000 (Haemonetics SA, IL, USA). TEG instruments were tested for quality control using standardized samples provided by the manufacturer. These results were always within range during the whole study period. TEG assessment protocol for r-TEG was as follows: 340 μL of sodium‑citrated whole blood and 20 μL of 0.2 mol/L of CaCl2 were used. The TEG cup contained 10 µL of reconstituted r-TEG reagent. No sample incubation was done before TEG analysis. Disposable cups were placed in the cup wells that were set at a temperature of 37°C. Normal ranges of various TEG parameters as per manufacturer’s protocol are as follows: R-time, two to eight minutes; K-time, one to three minutes; alpha angle, 55°-78°; maximal amplitude (MA), 51-69 mm; activated clotting time (ACT), 80-118 sec; clotting index (CI), −3 to 3, and lysis at 30 minutes (LY30), 0-8%. The hemostatic condition was defined as hypocoagulable if two or more of the following parameters were observed: increased R-time, increased K-time, decreased alpha angle, and/or decreased MA, and hypercoagulable if two or more of the following parameters were observed: decreased R-time, decreased K-time, increased alpha angle, and/or increased MA. LY30 measures percent lysis 30 min after MA and was used to diagnose either primary or secondary fibrinolysis. Primary fibrinolysis was defined when LY30 was higher than the upper limit of the normal reference range, with CI below the lower limit of the normal reference range. Secondary fibrinolysis was defined when LY30 and CI were higher than the upper limit of the normal reference range [17].

Statistical analysis

Data was analyzed using Stata statistical software, version 15 (StataCorp 2017, College Station, TX, USA) and RStudio version 2024.12.0+567 Posit software. Data were expressed as mean ± SD and median (min-max). The continuous variables and categorical variables were analyzed between groups using Fisher’s exact test, Wilcoxon rank sum test, and Pearson’s chi-squared test, as applicable. The Pearson correlation coefficient, denoted by “r,” was used to assess the correlation between r-TEG parameters and CCT using a heat map. Area under the receiver operating characteristics (AUROC) curve analysis was performed for r-TEG measurements and CCT to assess their predictive performance for mortality. Hazard ratio (unadjusted and age-adjusted) was calculated between survivors and non-survivors. The unadjusted and age-adjusted hazard ratios for mortality based on patient characteristics were derived using the Cox proportional hazards model.

## Results

Patient characteristics

The patient characteristics of the whole cohort, normal, hypocoagulable, and hypercoagulable states of the 105 patients with sTBI are illustrated in Table 1.

**Table 1:** Patient characteristics of the whole cohort, normal, hypocoagulable, and hypercoagulable states ^a^Fisher’s exact test; ^b^Wilcoxon rank sum test. All values are in median (IQR). IQR, interquartile range; ISS, Injury Severity Score; GCS, Glasgow Coma Scale; PR, pulse rate; RR, respiratory rate; SBP, systolic blood pressure; DBP, diastolic blood pressure; RTA, road traffic accident; SDH, subdural hemorrhage; SAH, subarachnoid hemorrhage; EDH, extradural hemorrhage; IPH, intraparenchymal hemorrhage

Characteristics	Whole cohort, n = 105	Normal, n = 61 (58%)	Hypocoagulable, n = 38 (36%)	Hypercoagulable, n = 6 (6%)	p-value
Age, years (IQR)	33 (25-40)	35 (26-40)	33 (25-40)	34 (28-45)	0.99^b^
Male, n (%)	96 (91)	58 (95)	33 (87)	5 (83)	0.22^a^
Female, n (%)	9 (9)	3 (5)	5 (13)	1 (17)	0.22^a^
ISS	29 (24-75)	29 (24-75)	57 (24-75)	52 (25-75)	0.40^b^
GCS	6 (4-7)	6 (4-7)	6 (4-7)	7 (7-7)	0.18^b^
PR (min)	90 (78-102)	88 (78-100)	96 (78-108)	92 (81-108)	0.50^b^
RR (min)	18 (18-20)	18 (18-20)	18 (18-20)	18 (16.50-18)	0.24^b^
SBP (mmHg)	123 (110-133)	124 (114-134)	114 (103-130)	145 (139-152)	<0.001^b^
DBP (mmHg)	76 (69-83)	76 (69-83)	74 (65-78)	90 (72-108)	0.07^b^
Length of stay (days)	6 (3-13)	6 (3-13)	5 (3-14)	5 (4-9)	0.89^b^
Mechanism of injury					
RTA, n (%)	79 (75)	47 (77)	27 (71)	5 (83)	0.73^a^
Fall, n (%)	15 (14)	9 (15)	6 (16)	0 (0)	0.73^a^
Unknown, n (%)	8 (8)	3 (5)	4 (11)	1 (17)	0.73^a^
Assault, n (%)	3 (3)	2 (3)	1 (2)	0 (0)	0.73^a^
Type of injury					
SDH, n (%)	45 (43)	25 (41)	17 (45)	3 (50)	0.39^a^
IPH, n (%)	33 (31)	23(37)	7 (18)	3 (50)	0.12^a^
SAH, n (%)	19 (18)	9 (15)	10 (26)	0 (0)	0.24^a^
EDH, n (%)	8 (8)	4 (7)	4 (11)	0 (0)	0.09^a^
Outcome, n (%)					
Discharge, n (%)	67 (64)	42 (63)	22 (33)	3 (4)	0.39^a^
Death, n (%)	38 (36)	19 (50)	16 (42)	3 (8)	0.39^a^

**Figure 1:**
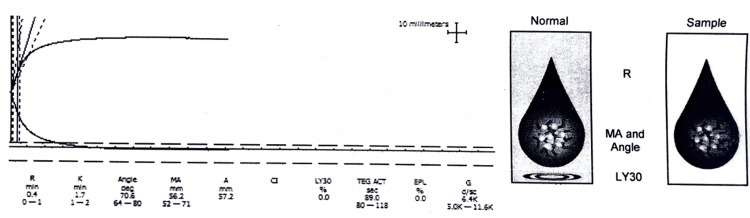
Rapid thromboelastography (r-TEG) graph of a patient (x-axis is time; the y-axis is millimeters of deviation representing increasing viscoelasticity of the sample) showing a normal pattern

The median (IQR) age of the whole cohort was 33 (25-40), and 91% were males. The most common mechanism of injury was road traffic accident (RTA) in 79 (75%) patients, followed by fall in 15 (14%), and was unknown in eight (8%) patients, and assault was present in three (3%) cases. The majority of patients had a subdural hematoma (43%), followed by intraparenchymal hemorrhage (31%), subarachnoid hemorrhage (18%), and extradural hemorrhage (8%). The hemostatic condition was categorized as normal in 61 (58%) (Figure 1).

**Figure 2:**
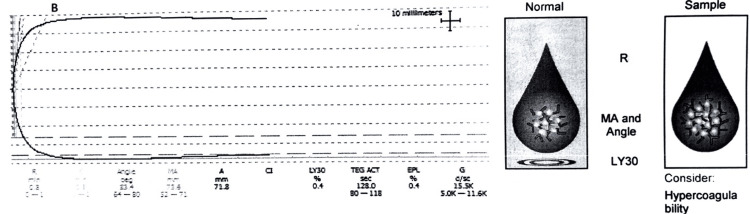
Rapid thromboelastography (r-TEG) graph of a patient (x-axis is time; the y-axis is millimeters of deviation representing increasing viscoelasticity of the sample) showing a hypercoagulability pattern

The r-TEG pattern showed a hypercoagulable state in six (6%) (Figure 2). 

**Figure 3:**
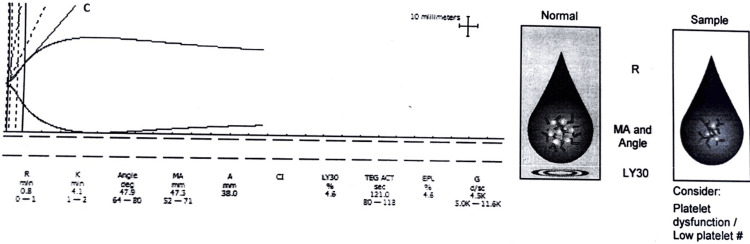
Rapid thromboelastography (r-TEG) graph of a patient (x-axis is time; the y-axis is millimeters of deviation representing increasing viscoelasticity of the sample) showing a hypocoagulability pattern

The r-TEG pattern showed hypocoagulable state in 38 (36%) (Figure 3).

The overall ISS score was 29 (24-75); maximum in patients with hypocoagulable state [57 (24-75)], followed by hypercoagulable [52 (25-75)], and lowest in patients with normal coagulation state [29 (24-75)]. The median GCS was 6 (4-7). Systolic blood pressure (SBP) and diastolic blood pressure (DBP) of the whole cohort were 123 (110-133) and 76 (69-83) mmHg, respectively; SBP was statistically significant among the three groups (p < 0.001). The mortality rate of the whole cohort was 36.2% (38/105). Among the patients who died 8% (3/38) were hypercoagulable, and 42% (16/38) were hypocoagulable, while 50% (19/38) were normal on r-TEG. 

Laboratory results

The laboratory results of all the groups are presented in Table 2.

**Table 2:** Laboratory parameters of the whole cohort, normal, hypocoagulable, and hypercoagulable groups ^a^Fisher’s exact test; ^b^Wilcoxon rank sum test. All values are in median (IQR). WBC, white blood cells

Parameters	Whole cohort (n = 105)	Normal (n = 61)	Hypocoagulable (n = 38)	Hypercoagulable (n = 6)	p-value
Hemoglobin, g/dL	13.20 (10.90-14.75)	13.10 (10.70-14.50)	13.45 (10.98-14.50)	14.05 (12.18-15.63)	0.28^b^
WBC, ×10^9^/L	15 (11-19)	14 (11-18)	17 (11-20)	24 (15-25)	0.10^b^
Platelet count, ×10^9^/L	199 (142-269)	203 (150-270)	154 (114-246)	232 (207-372)	0.06^b^
Urea, mg/dL	25 (20-34)	25 (21-34)	25 (20-34)	22 (19-28)	0.71^b^
Creatinine, mg/dL	0.80 (0.70-1.00)	0.80 (0.70-0.99)	0.80 (0.70-1.10)	0.73 (0.70-0.79)	0.56^b^
Sodium, mEq/L	140.0 (138.0-143.0)	140.0 (138.0-143.5)	139.0 (135.0-141.0)	140.2 (139.0-143.4)	0.08^b^
Potassium, mEq/L	4.06 (3.68-4.50)	4.10 (3.79-4.52)	3.97 (3.45-4.39)	3.91 (3.53-4.20)	0.51^b^
Chloride, mEq/L	108.0 (104.0-110.0)	109.0 (106.0-111.5)	107.4 (102.3-109.5)	103.0 (100.0-107.8)	0.04^b^
Total bilirubin, mg/dL	0.80 (0.50-1.02)	0.80 (0.50-0.96)	0.75 (0.46-1.20)	0.95 (0.80-1.71)	0.25^b^
Blood glucose, mg/dL	146 (118-186)	137 (115-165)	167 (139-214)	120 (116-145)	0.03^b^

Among the laboratory parameters, serum chloride and blood sugar were found to be statistically significant between all the groups (p = 0.04 and p = 0.03, respectively).

Coagulation profile and TEG analysis

The coagulation profile, including the TEG analysis of all groups, is shown in Table 3.

**Table 3:** Coagulation profile including TEG data of the whole cohort, normal, hypocoagulable, and hypercoagulable states ^a^Fisher’s exact test; ^b^Wilcoxon rank sum test PT, prothrombin time; INR, international normalized ratio; aPTT, activated partial thromboplastin time; MA, maximum amplitude; TPI, thrombodynamic potential index; E, elasticity constant; G, shear elastic modulus strength; TMA, time to maximum amplitude; ACT, activated clotting time; LY30-lysis at 30 minute; A, amplitude

Parameter	Total (n = 105)	Normal (n = 61)	Hypocoagulable (n = 38)	Hypercoagulable (n = 6)	p-value
PT, sec	14.5 (13.1-16.2)	14.20 (12.9-16.5)	14.8 (14.03-16.03)	13.5 (11.83-14.35)	0.13^b^
INR	1.17 (1.06-1.3)	1.16 (1.05-1.3)	1.23 (1.12-1.3)	1.08 (0.99-1.24)	0.18^b^
aPTT, sec	28 (25-31)	28 (25-30)	30 (27-33)	28 (27-31)	0.03^b^
Fibrinogen, mg/dL	314 (204-440)	331 (218-440)	234 (167-390)	436 (383-647)	0.007^b^
D-dimer, ng/mL (DDU)	2,402 (1,050-5,250)	2,241 (1,050-5,250)	3,430 (1,050-5,250)	2,289 (1,303-4,566)	0.73^b^
R-time (min)	0.7 (0.4-0.9)	0.6 (0.4-0.8)	0.8 (0.4-1.35)	0.5 (0.33-0.75)	0.04^b^
K-time (min)	1.80 (1.3-2.4)	1.6 (1.2-1.8)	2.9 (2.3-4.1)	0.8 (0.8-0.8)	<0.001^b^
Alpha angle (deg.)	69 (62-74)	72 (69-75)	57 (49-63)	81 (80-83)	<0.001^b^
MA, mm	61 (54-65)	63 (61-65)	49 (46-54)	75 (71-75)	<0.001^b^
TPI	41 (22-69)	52 (43-74)	19 (9-25)	181 (146-185)	<0.001^b^
TMA, min	20.9 (18.6-22.9)	20.2 (18.4-21.2)	23.2 (21.0-25.0)	16.8 (16.5-17.6)	<0.001^b^
G, dyne/sec	7,651 (5,260-9,088)	8,372 (7,651-9,267)	4,781 (4,047-5,843)	15,101 (12,129-15,424)	<0.001^b^
E, dyne/sec	153 (105-182)	167 (153-185)	96 (81-117)	302 (243-308)	<0.001^b^
ACT, sec	113 (89-136)	105 (89-121)	121 (89-158)	97 (84-122)	0.13^b^
LY30, %	0.9 ± 9.3	0.02 ± 0.5	2.73 ± 15.4	0.05 ± 0.2	0.37^b^
A, mm	60 (53-64)	62 (59-64)	50 (45-55)	73 (70-76)	<0.001^b^

Among all the CCT, aPTT and fibrinogen were found to be statistically significant between all the groups (p = 0.03 and p = 0.007, respectively). Among TEG parameters, R-time, K-time, alpha angle, MA, TPI, TMA, G, E, and A were all found to be statistically significant between the groups.

Comparative evaluation of variables between survivors and non-survivors

Comparative analysis of variables among survivors and non-survivors is shown in Table 4.

**Table 4:** Comparative analysis of variables among survivors and non-survivors ^a^Fisher’s exact test; ^b^Wilcoxon rank sum test; ^c^Pearson’s chi-squared test IQR, interquartile range; ISS, Injury Severity Score; GCS, Glasgow Coma Scale; PR, pulse rate; RR, respiratory rate; SBP, systolic blood pressure; DBP, diastolic blood pressure; RTA, road traffic accident; SDH, subdural hematoma; SAH, sub arachnoid hemorrhage; EDH, extradural hematoma; IPH, intraparenchymal hemorrhage; PT, prothrombin time; INR, international normalized ratio; aPTT, activated partial thromboplastin time; MA, maximum amplitude; TPI, thrombodynamic potential index; E, elasticity constant; G, shear elastic modulus strength; TMA, time to maximum amplitude; ACT, activated clotting time; LY30-lysis at 30 minute; A, amplitude

Variables	Survivor, n = 67 (64%)	Non-survivors, n = 38 (36%)	p-value
Age, years (mean, IQR)	32 (25-40)	35 (30-46)	0.1^b^
Male, n (%)	64 (96)	32 (84)	0.07^a^
Female, n (%)	3 (4)	6 (16)	0.07^a^
Mechanism of injury, n (%)			
RTA	55 (82)	24 (63)	0.03^a^
Assault	0 (0)	3 (7.9)	0.03^a^
Fall	7 (10)	8 (21)	0.03^a^
Unknown	5 (7.5)	3 (7.9)	0.03^a^
ISS	26 (22-29)	75 (75-75)	<0.001^b^
GCS	7.00 (5.50-7.00)	5.0 (3.25-6.0)	<0.001^b^
Length of stay (days)	6 (4-18)	4 (2-9)	0.001^b^
PR (per min)	89 (79-100)	90 (75-109)	0.76^b^
RR (per min)	18 (18-20)	18 (18-20)	0.28^b^
SBP, mmHg	123 (113-133)	121 (107-137)	0.47^b^
DBP, mmHg	75 (70-82)	76 (66-87)	0.73^b^
Hb, g/dL	13.5 (11.7-14.8)	12.30 (10.7-14.4)	0.28^b^
WBC, ×10^9^/L	15 (11-19)	15 (11-20)	0.84^b^
Platelet, ×10^9^/L	203 (147-277)	162 (116-243)	0.08^b^
Urea, mg/dL	26 (20-33)	25 (21-38)	0.89^b^
Creatinine, mg/dL	0.80 (0.70-0.99)	0.80 (0.7-1.1)	0.99^b^
Total bilirubin, mg/dL	0.70 (0.50-0.96)	0.86 (0.6-1.2)	0.16^b^
Blood sugar, mg/dL	137 (114-169)	162 (137-234)	0.01^b^
Sodium, mEq/L	140 (138.0-142)	139.5 (137-143)	0.80^b^
Potassium, mEq/L	4.01 (3.7-4.5)	4.20 (3.7-4.5)	0.70^b^
Chloride, mEq/L	109.0 (106-110)	107.8 (102-109)	0.14^b^
PT, min	14.2 (12.8-15.8)	14.9 (13.3-16.6)	0.20^b^
INR	1.13 (1.1-1.28)	1.24 (1.1-1.4)	0.01^b^
aPTT, min	28 (25-30)	30 (28-36)	0.001^b^
D-dimer, ng/mL (DDU)	2,004 (1,050-5,250)	4,375 (1,050-5,250)	0.09^b^
Fibrinogen, mg/dL	270 (202-406)	364 (237-577)	0.11^b^
R, min	0.60 (0.40-1.00)	0.70 (0.40-0.90)	0.51^b^
K, min	1.70 (1.25-2.25)	2.00 (1.40-2.90)	0.18^b^
Alpha angle (deg.)	70 (65-74)	68 (56-74)	0.20^b^
MA, mm	70 (65-74)	68 (56-74)	0.20^b^
G, dyne/sec	7,857 (5,899-9,181)	7,078 (4,245-8,587)	0.24^b^
TPI	46 (26-70)	33 (10-59)	0.16^b^
TMA	20.7 (18.7-22.4)	21.5 (18.6-23.1)	0.28^b^
E, dyne/sec	157 (118-184)	142 (85-172)	0.24^b^
LY30, %	0.17 ± 0.70	2.47 ± 15.46	0.01^b^
A, mm	60 (54-64)	59 (48-63)	0.42^b^
ACT, sec	105 (89-136)	113 (89-136)	0.34^b^

Unadjusted and age-adjusted hazard ratios for mortality based on patient characteristics

The unadjusted and age-adjusted hazard ratios for mortality based on patient characteristics are shown in Table 5.

**Table 5:** Unadjusted and age-adjusted hazard ratios for mortality based on patient characteristics (Cox proportional hazards model) HR, hazard ratio; CI, confidence interval; IQR, interquartile range; ISS, Injury Severity Score; GCS, Glasgow Coma Scale; PR, pulse rate; RR, respiratory rate; SBP, systolic blood pressure; DBP, diastolic blood pressure; RTA, road traffic accident; SDH, subdural hematoma; SAH, sub arachnoid hemorrhage; EDH, extradural hematoma; IPH, intraparenchymal hemorrhage; PT, prothrombin time; INR, international normalized ratio; aPTT, activated partial thromboplastin time; MA, maximum amplitude; TPI, thrombodynamic potential index; E, elasticity constant; G, shear elastic modulus strength; TMA, time to maximum amplitude; ACT, activated clotting time; LY30-lysis at 30 minute; A, amplitude

Characteristic	Unadjusted HR (95% CI)	p-value	Age-adjusted HR (95% CI)	p-value
Age, years (mean, IQR)	1.02 (1.0-1.04)	0.07	-	-
Male, n (%)	2.97 (1.2-7.2)	0.01	2.50 (0.99-6.3)	0.07
GCS	0.65 (0.5-0.8)	<0.001	0.66 (0.5-0.8)	<0.001
PR (per min)	1.0 (0.9-1.0)	0.72	1.0 (0.9-1.02)	0.79
RR (per min)	0.97 (0.9-1.1)	0.42	0.97 (0.9-1.1)	0.38
SBP, mmHg	0.99 (0.9-1.0)	0.11	0.98 (0.9-1.0)	0.03
DBP, mmHg	0.99 (0.9-1.0)	0.38	0.98 (0.9-1.0)	0.24
Hb, g/dL	0.91 (0.8-1.0)	0.14	0.92 (0.8-1.0)	0.19
WBC, ×10^9^/L	1.02 (0.9-1.0)	0.47	1.03 (0.9-1.1)	0.32
Platelet, ×10^9^/L	1.0 (0.9-1.0)	0.14	1.0 (0.9-1.0)	0.13
Urea, mg/dL	1.0 (0.9-1.0)	0.59	0.99 (0.9-1.0)	0.30
Creatinine, mg/dL	1.07 (0.7-1.6)	0.75	0.97 (0.6-1.5)	0.88
Total bilirubin, mg/dL	1.04 (0.7-1.5)	0.84	0.99 (0.7-1.4)	0.97
Blood sugar, mg/dL	1.01 (1.0-1.0)	<0.001	1.01 (1.0-1.0)	<0.001
Sodium, mEq/L	1.02 (0.9-1.1)	0.47	1.02 (0.9-1.9)	0.52
Potassium, mEq/L	1.16 (0.8-1.8)	0.48	1.15 (0.8-1.8)	0.53
Chloride, mEq/L	0.92 (0.8-1.0)	0.07	0.92 (0.8-1.0)	0.07
PT, min	1.02 (1.0-1.0)	0.008	1.01 (1.0-1.0)	0.09
INR	1.23 (1.1-1.4)	0.004	1.19 (1.0-1.4)	0.07
aPTT, min	1.0 (1.0-1.0)	<0.001	1.01 (1.0-1.02)	0.02
D-dimer, ng/mL (DDU)	1.0 (1.0-1.0)	0.16	1.0 (1.0-1.0)	0.21
Fibrinogen, mg/dL	1.0 (1.0-1.0)	0.04	1.0 (1.0-1.0)	0.03
R, min	2.22 (1.20-4.12)	0.01	2.11 (1.14-3.94)	0.03
K, min	1.11 (1.02-1.20)	0.01	1.11 (1.02-1.20)	0.04
Alpha angle (deg.)	0.96 (0.94-0.99)	0.004	0.97 (0.94-0.99)	0.01
MA, mm	0.97 (0.95-1.00)	0.06	0.97 (0.95-1.00)	0.08
G, dyne/sec	1.0 (1.0-1.0)	0.87	1.0 (1.0-1.0)	0.36
TPI	1.00 (0.99-1.0)	0.58	1.00 (0.99-1.0)	0.91
TMA	0.99 (0.95-1.0)	0.37	0.98 (0.95-1.0)	0.45
E, dyne/sec	1.00 (0.99-1.0)	0.37	1.0 (0.99-1.0)	0.36
LY30, %	0.67 (0.41-1.1)	0.10	0.62 (0.37-1.0)	0.07
A, mm	0.98 (0.95-1.0)	0.09	0.98 (0.95-1.0)	0.11
ACT, sec	1.01 (1.0-1.0)	0.005	1.01 (1.0-1.0)	0.02

Outcome

**Figure 4:**
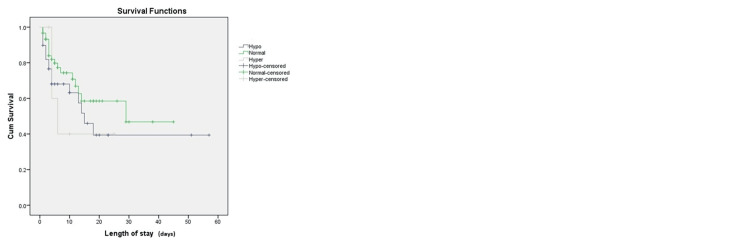
Survival probability among patients with different coagulation status The Kaplan-Meier Survival analysis curve for all the groups. The 57-day mortality among patients with hypocoagulable and hypercoagulable was higher than that of patients with normal coagulation status (log-rank test, p = 0.35).

At a median follow-up of six days (IQR, 1-57), 38 (36%) patients died. Thirty-four of them died due to cardiac arrest, two died due to raised intracranial tension due to severe head trauma, and one each died of hemorrhagic shock and acute respiratory distress syndrome. The 57-day mortality among patients with hypocoagulable and hypercoagulable was higher than that of those with normal coagulation status (log-rank test, p = 0.35; Figure 4).

**Figure 5:**
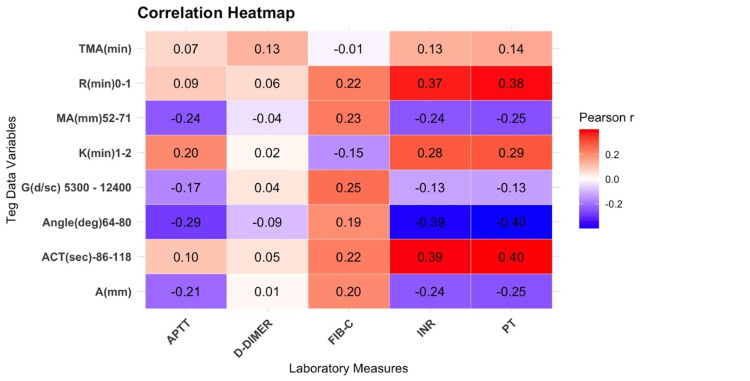
Heat map showing comparison of TEG parameters to the CCT CCT, conventional clotting tests; TEG, thromboelastography

Heat map for the comparison of TEG parameters to the CCT shows positive correlation of R-time to PT and INR (r = 0.38 and 0.37, respectively), whereas alpha angle was inversely correlated to PT and INR (r = −0.40 and −0.39, respectively) (Figure 5).

**Figure 6:**
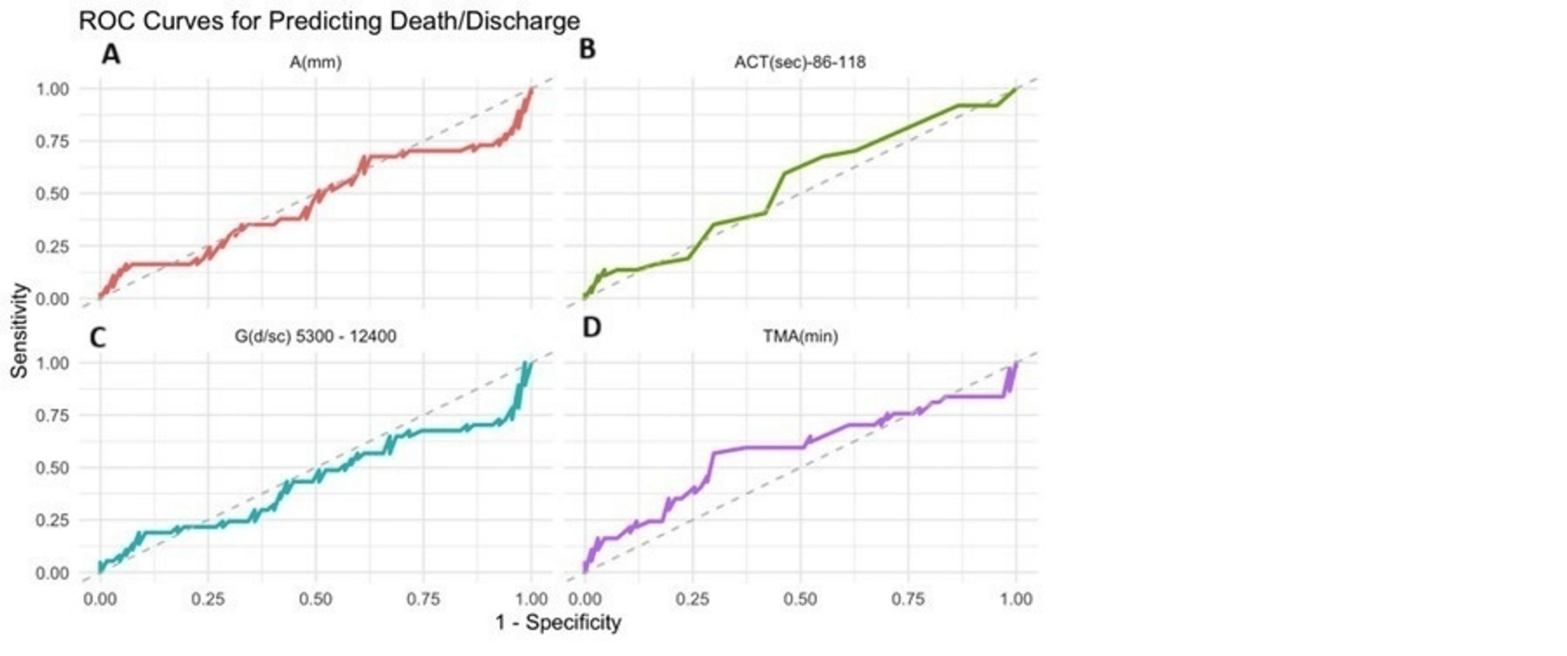
ROC curve showing outcome with r-TEG parameters (A) AUC for A (amplitude) is 0.46 with a sensitivity of 0.16 and a specificity of 0.94. (B) AUC for ACT is 0.54 with a sensitivity of 0.59 and a specificity of 0.54. (C) AUC for G is 0.44 with a sensitivity of 0.19 and a specificity of 0.91. (D) AUC for TMA is 0.58 with a sensitivity of 0.57 and a specificity of 0.70. ROC, receiver operating characteristic curve; r-TEG, rapid thromboelastography; AUC, area under the curve; ACT, activated clotting time; TMA, time to maximum amplitude; G, shear elastic modulus

The receiver operating characteristic curve (ROC) is shown for predicting the outcome with regard to the r-TEG parameters in Figures 6A-6D.

**Figure 7:**
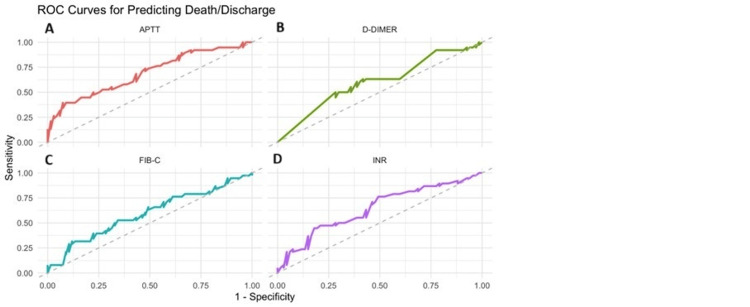
ROC curve showing outcome with CCT (A) AUC for aPTT is 0.69 with a sensitivity of 0.76 and a specificity of 0.50. (B) AUC for D-dimer is 0.59 with a sensitivity of 0.60 and a specificity of 0.61. (C) AUC for fibrinogen-C is 0.59 with a sensitivity of 0.31 and a specificity of 0.88. (D) AUC for INR is 0.64 with a sensitivity of 0.76 and a specificity of 0.50. CCT, conventional clotting tests; ROC, receiver operating characteristic curve; AUC, area under the curve; INR, international normalized ratio

Similarly, the ROC curve is shown for predicting the outcome with regard to the CCT parameters in Figures 7A-7D.

## Discussion

TEG is a commonly used VHA in trauma settings. It is a point-of-care test that quantitatively evaluates the clotting process of whole blood. TEG is particularly more sensitive in detecting abnormalities in clot strength, fibrinolysis, and platelet function, components often overlooked by routine assays like PT, aPTT, and INR in patients with TBI [17]. CCT are neither able to identify a hypercoagulable state in vitro nor provide a functional assessment of hemostatic mechanisms in vivo. TEG may be used as a global assessment of coagulation, permitting the diagnosis of hypercoagulable and hypocoagulable states based on the viscoelastic properties of blood [18]. 

In our study, we were able to categorize sTBI patients into normal coagulation, hypocoagulable, and hypercoagulable states based on r-TEG and compared the coagulation status to the outcome of these patients. We identified coagulopathic states after TBI, defined by the r-TEG parameters such as R-time, K-time, alpha angle, MA, TMA, G, TPI, E, and A. The hypercoagulable state was characterized by a decrease in R-time, K-time, and TMA, while an increase in alpha angle, MA, TMA, G, TPI, E, and A. The hypocoagulable state was characterized by an increase in R-time, K-time, and TMA, while a decrease in alpha angle, MA, TMA, G, TPI, E, and A. The hypercoagulable state may represent platelet hyperactivity after TBI, as MA represents clot strength and is the end result of maximal platelet-fibrin interaction via the GP IIb-IIIa receptors, forming the platelet plug [19]. Clot strength consists primarily of platelet function (approximately 80%) and fibrinogen activity (20%) [20]. The G value is a calculated parameter that reflects the complete strength of the clot from initial thrombin generation through fibrinolysis. The G value is a more sensitive measure of platelet function and shares an exponential relationship with MA [19]. Isolated TBI is often associated with abnormalities in coagulation parameters. Meta-analysis of 34 studies reporting the frequencies of coagulopathy after TBI showed an overall prevalence of 32.7% [21]. The presence of coagulopathy after TBI was related both to mortality and unfavorable outcome [21]. In our study, the presence of coagulopathy was seen in 42% of TBI patients, with mortality in 36% cases. The presence of coagulation disorder has been linked to the progression of both hemorrhagic and ischemic lesions and is associated with increases in morbidity and mortality. The mechanisms underlying coagulopathy after TBI are still poorly understood. Current evidence suggests that it is a dynamic process involving a state of hypercoagulability followed by a bleeding diathesis. The most commonly accepted hypothesis of the pathogenesis of coagulopathy after TBI implies alterations in local and systemic coagulation and fibrinolytic pathways secondary to the release of TF, disseminated intravascular coagulation, platelet dysfunction, and activation of protein C pathways secondary to hypoperfusion [22].

Current evidence suggests that disruption of the coagulation cascade caused by TBI creates both a hypocoagulable and hypercoagulable state [23]. In our study, we found 36% of the patients succumbed, of which 50% had coagulopathy. In this study, we found that r-TEG parameters such as R-time, K-time, alpha (ɑ) angle, and ACT were statistically significant and associated with mortality. Holcomb et al. showed that major trauma activations that r-TEG parameters, except for G value, were associated with 24-hour and 30-day mortality [24]. Using r-TEG, Kashuk et al. identified 67% of a cohort of 152 critically ill patients in the surgical intensive care unit (ICU) for seven months to be hypercoagulable based on G score [25]. The identified hypercoagulable state was predictive of thromboembolic events, and no patient with a normal coagulation profile had a thrombotic event [25]. In this study, we identified 6% patients with sTBI with hypercoagulability using two or more r-TEG parameters, such as decreased R-time, decreased K-time, increased alpha angle, and/or increased MA. Kunio et al. identified hypocoagulability in 9% of 69 TBI patients, which correlated with an increase in mortality [26]. They concluded that TEG-defined hypocoagulable profiles on admission have been associated with worse outcomes and increased need for neurosurgical interventions [27]. In our study, the hemostatic condition was categorized as hypocoagulable in 36% patients. Hypocoagulability was associated with poor prognosis. Among TEG parameters, R-time, K-time, alpha angle, MA, TPI, TMA, G, E, and A were found to be statistically significant between all the groups.

Limitations

These results must be interpreted in light of a few limitations of this study. Despite the demonstration of a strong association between r-TEG and coagulopathy among patients with sTBI, the investigators could not evaluate the relationship between r-TEG and transfusion requirements. Also, further studies are required to establish the relationship between coagulopathy and sTBI.

## Conclusions

r-TEG is a valuable tool for assessing coagulation abnormalities in patients with sTBI. In this study, r-TEG was utilized to detect coagulopathy early in the clinical course, allowing for a more comprehensive evaluation of the hemostatic profile beyond conventional laboratory tests. A significant proportion of sTBI patients demonstrated r-TEG-defined coagulopathy, which was associated with markedly increased mortality rates. Patients with coagulopathic profiles had poorer outcomes compared to those with normal coagulation, highlighting the prognostic significance of early coagulation assessment. These findings suggest that r-TEG can play a crucial role in identifying high-risk patients and guiding timely hemostatic interventions to potentially improve survival in sTBI.
